# Inter-Hospital Variability of Postoperative Pain after Tonsillectomy: Prospective Registry-Based Multicentre Cohort Study

**DOI:** 10.1371/journal.pone.0154155

**Published:** 2016-04-27

**Authors:** Orlando Guntinas-Lichius, Katharina Geißler, Marcus Komann, Peter Schlattmann, Winfried Meissner

**Affiliations:** 1 Department of Otorhinolaryngology, Jena University Hospital, Jena, Germany; 2 Department of Anaesthesiology and Intensive Care Medicine, Jena University Hospital, Jena, Germany; 3 Department of Medical Statistics, Computer Sciences and Documentation, Jena University Hospital, Jena, Jena, Germany; Scientific Inst. S. Raffaele Hosp., ITALY

## Abstract

**Objectives:**

Although tonsillectomy is one of the most frequent and painful surgeries, the association between baseline and process parameters and postoperative pain are not fully understood.

**Methods:**

A multicentre prospective cohort study using a web-based registry enrolled 1,527 women and 1,008 men aged 4 to 85 years from 52 German hospitals between 2006 and 2015. Maximal pain (MP) score the first day after surgery on a numeric rating scale (NRS) from 0 (no pain) to 10 (MP) was the main outcome parameter.

**Results:**

The mean maximal pain score was 5.8±2.2 (median 6). Multivariable analysis revealed that female gender (Odds ratio [OR] = 1.33; 95% confidence interval [CI] = 1.12 to 1.56; p = 0.001), age <20 years (OR = 1.56; CI = 1.27 to 1.91; p<0.0001), no pain counselling (OR = 1.78; CI = 1.370 to 2.316; p<0.001), chronic pain (OR = 1.34; CI = 1.107 to 1.64; p = 0.004), and receiving opioids in recovery room (OR = 1.89; CI = 1.55 to 2.325; p<0.001) or on ward (OR = 1.79; CI = 1.42 to 2.27; p<0.001) were independently associated with higher experienced maximal postoperative pain (greater the median of 6). The effect of age on pain was not linear. Maximal pain increased in underage patients to a peak at the age of 18 to 20 years. From the age of ≥20 years on, maximal pain after tonsillectomy continuously decreased. Even after adjustment to all statistically important baseline and process parameters, there was substantial variability of maximal pain between hospitals with a heterogeneity variance of 0.31.

**Conclusion:**

Many patients seem to receive insufficient or ineffective analgesia after tonsillectomy. Further research should address if populations at risk of higher postoperative pain such as females, younger patients or those with preexisting pain might profit from a special pain management protocol. Beyond classical demographical and process parameters the large variability between different hospitals is striking and indicates the existence of other unknown factors influencing postoperative pain after tonsillectomy.

## Introduction

Although tonsillotomy, i.e. the partial surgical removal of the palatine tonsils, has nowadays replaced some indications of tonsillectomy, i.e. the complete surgical removal of the palatine tonsils, tonsillectomy still is one of the most common surgical procedures in children and adults. For instance, 737,000 tonsillectomies were performed in the United States in 2006 [1]. 39.262 and 54,441 tonsillectomies were performed in 2014 in the United Kingdom and in Germany, respectively [2,3]. Tonsillectomy causes severe postoperative pain lasting for many days [4]. Recently, a prospective cohort study using data of 70,764 patients taking part in the Quality Improvement in Postoperative Pain Treatment (QUIPS) registry has shown that tonsillectomy, although classified as a so called minor procedure, was one of the most painful surgical procedures [5]. The authors concluded that patients who undergo minor surgery perhaps are given less analgesia than needed. Two recent German studies with relative small sample size confirmed that postoperative pain is relevant but pain management may be is insufficient and needs improvement after tonsillectomy [6–8]. Nevertheless, not much is known about predictors for increased postoperative pain after tonsillectomy and there is no international standard pain therapy regime for children or adults after tonsillectomy.

The above mentioned project QUIPS was developed in 2005, consisting of standardized data acquisition and an analysis of quality and process indicators [9]. QUIPS, its corresponding project in children QUIPSI and the international counterpart PAIN OUT are open for every hospital worldwide and are web-based [7,10,11]. Currently, more than 200 hospitals are participating and more than 430,000 patients have been included. An analysis of the first 22,963 included patients has shown that preoperative chronic pain, younger age and female gender were predictors for higher postoperative pain [12].

A detailed analysis for pain predictors in patients who underwent tonsillectomy has not yet been performed. Hence, the present prospective clinical study used QUIPS data to analyse 1) postoperative pain within the first 24 hours in children and adults after tonsillectomy, 2) its pain management, and 3) associated parameters.

## Methods

### Pain and pain management measures

The present prospective cohort study was part of the German-wide Quality Improvement in Postoperative Pain Treatment (QUIPS) registry and the Quality Improvement in Postoperative Pain Treatment in Children (QUIPSInfant) registry. Institutional review board approval was obtained prior to study initiation by the Ethics Committee of the Jena University Hospital, Thuringia, Germany. The QUIPS and QUIPSInfant questionnaires are presented in detail elsewhere [7,13]. The QUIPS and QUIPSInfant questionnaires consisted of two parts for each patient: This first part is covering the patient-reported outcome parameters of the questionnaire, whereas the second part, which is filled by the investigator, addresses demographic and clinical data.

Patients and, if appropriate, the parents were informed in written form as well as orally by the study personnel. Informed consent was documented by filling in the questionnaire. The patients received a validated 15-item QUIPS or QUIPSInfant questionnaire at the first postoperative day. After a standardized instruction, the patient him-/herself completed the part one of the form. In children, a standardized instruction to the parents and the child was given. If the child was too young to read the questions himself, the questions were read to him. The patients filled out the questionnaire back on ward at the first postoperative day, i.e. 24–30 hours after surgery. Each patient was questioned only once. The time span was depending on exact time of surgery the day before (start of surgery: 7 a.m. to 3 p.m.). QUIPS used 11-point numeric rating scales (NRS) to estimate the patient’s pain during movement, maximal pain and pain at rest. Generally, higher numbers are indicating more pain (0 = no pain; 10 = maximal pain). QUIPSI used Faces from the Faces Pain Scale-Revised from Hicks et al. [14] to estimate postoperative pain in three above mentioned pain categories. Generally, from left to right, the faces and higher numbers indicate more pain. Furthermore, the patient was asked by dichotomized (yes/no) questions about pain-related impairments (mobility, breathing, sleep, mood), side effects of pain treatment (drowsiness, nausea, vomiting), and satisfaction with the pain management. The patients were also asked about the preoperative pain counselling in three categories (yes, in general; yes, specific; no). General pain counselling meant that education about postoperative pain and its management in general was part of the pre-surgical interview with the patients. Specific pain counselling assumed that it was talked about specific pain issues related to the respective surgical procedure. Furthermore, the interview had to include education on specific measures to prevent and manage postoperative pain before, during and after tonsillectomy for the individual patient.

**Fig 1:**
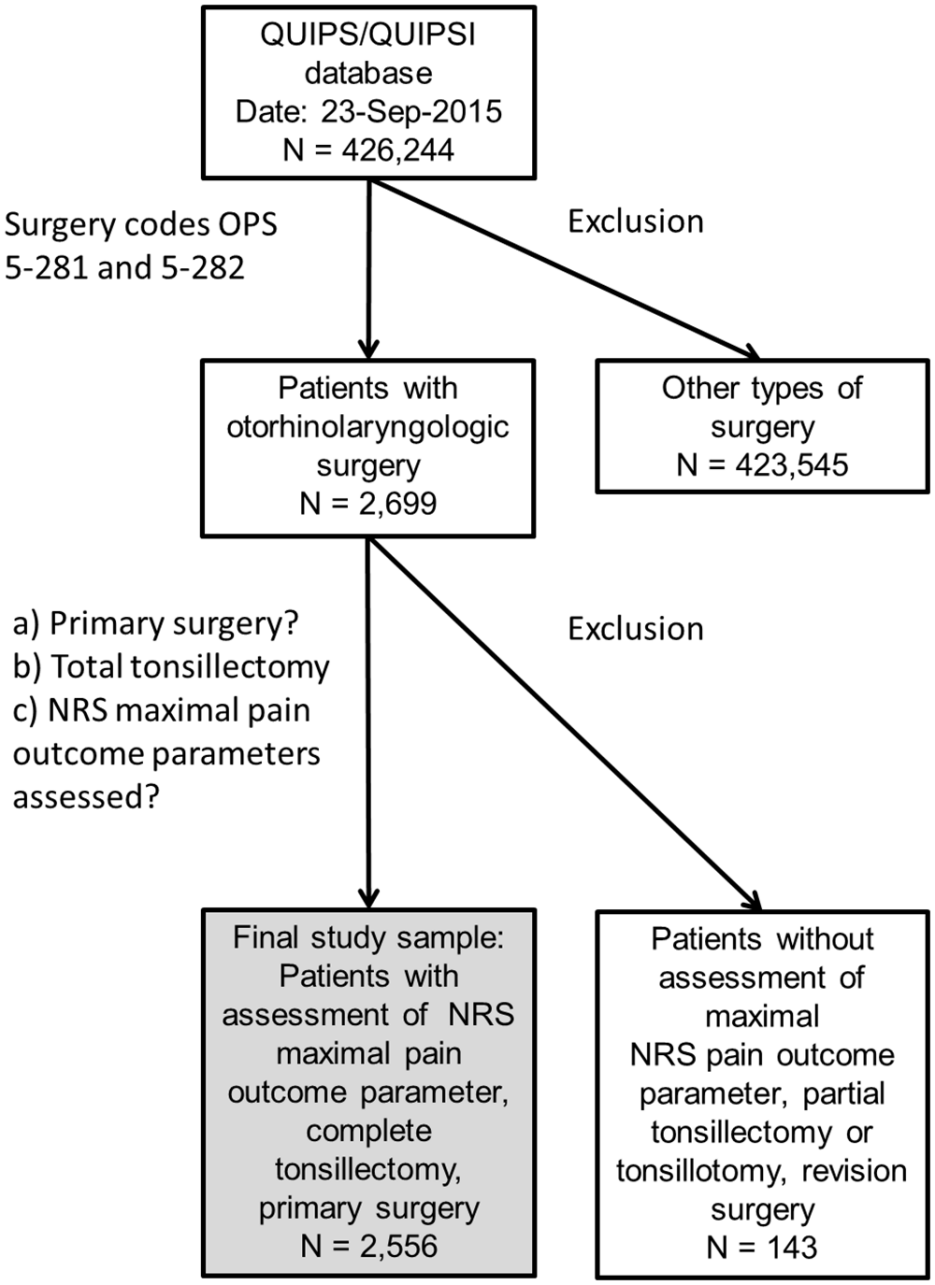
Flowchart of inclusion and exclusion criteria. NRS = numeric rating scale; OPS = German Surgical Procedure Coding; QUIPS = Quality Improvement in Postoperative Pain Treatment; QUIPSI = Quality Improvement in Postoperative Pain Treatment in Infants.

The second part of the questionnaires, which is filled by the investigator, was identical for QUIPS and QUIPSInfant and covers the relevant demographic and clinical parameters like age, gender, type of surgery, anaesthesia, and pain management. Only pain therapy measures applied before the patient filled out the questionnaire were registered. All data was anonymised and transferred via internet (http://www.quips-projekt.de/) to the QUIPS database allowing an anonymous comparison to the results of other participating hospitals (Procedure codes in S1 Table). A databank manager inspected all collected data for inconsistencies and, completeness, and eliminated erroneous or inconsistent values. This step transformed all raw data into validated data. In the participating hospitals, all adult patients and children >4 years scheduled for tonsillectomy could be included into the QUIPS or QUIPSInfant project. Exclusion criteria were as follows: age < 4 years; patient was transferred to another ward after surgery; patient or parents refused participation or had been discharged before data collection on the first postoperative day. Numbers and reasons for exclusion of patients are presented in Fig 1.

### Subjects

All patients participating in QUIPS and QUIPSInfant between November 2006 and September 2015 were included in the analysis. By German law and reimbursement requirements, the German procedure classification (OPS-301) has to be used by all German hospitals for coding of surgical procedures (OPS codes). To select all patients with tonsillectomy, first all patients with the OPS codes 5–281 (tonsillectomy without adenoidectomy) and 5–282 (tonsillectomy with adenoidectomy) were selected. Patients with the OPS codes 5–281.4 (surgery of tonsil remnants) and 5–281.5 (partial tonsillectomy/tonsillotomy) were excluded. Only patients receiving surgery with cold instruments were included. Other tonsillectomy techniques were excluded. Furthermore, only patients were included when of the three NRS pain outcome measures at least the maximal pain score was available. Some of the patients were already included in a recent study comparing postoperative pain between 179 surgical procedures [5] or in a study comparing different otorhinolaryngological surgeries [7,8].

### Statistical analysis

Data is presented as mean ± standard deviation (SD) if not otherwise indicated. Clinical and outcome parameters of all patients were summarized descriptively. Associations among patients`characteristics and clinical parameters, respectively, and maximal pain were examined via chi-square test. Correlations between pain during movement, maximal pain and minimal pain were analysed by Spearman’s correlation. Multivariable ordinal regression with random intercepts for hospitals was used for the statistical analysis of the primary outcome parameter maximal pain. This model accounts for variability between hospitals [15]. Variable selection was based on a two-step approach. Patients’ characteristics and clinical parameters known from the literature were derived from those suggestive for statistically important associations (p<0.20) [16]. In a second step model selection was based on the likelihood ratio test using stepwise entry. Multivariable binary logistic regression models with stepwise entry were used for the dichotomized categorized outcome parameter maximal pain to analyse the association to pain-related interference and pain therapy side effectsIn general, nominal p values of two-tailed tests are reported. In order to analyse the functional form of the beyond a linear relationship between age and pain intensity fractional polynomials were applied [17]. The significance level was set at p<0.05. Descriptive analysis of patients’ characteristics and QUIPS/QUIPSInfant variables were performed with IBM SPSS statistics software (Version 23.0.0.0). Fractional polynomials were estimated with STATA 14. Ordinal logistic regression with random effects was performed with SAS 9.4, proc glimmix.

## Results

### Subjects

During the study recruitment interval 426,244 patients participated in QUIPS and QUIPSI (Fig 1). A total of 2,699 patients with tonsil surgery were identified. Finally, 2,556 patients who had undergone primary tonsillectomy with assessment of at least the maximal NRS pain outcome were eligible for further analysis. The group of included participants is presented in Table 1. There was a female predominance (60%). Most patients were adult (85%). 63% of the patients were ≤25 years old. Half of the patients had an ASA status 1 and four of five patients an ASA status ≤2. 20% reported of chronic pain of other origin independent of the actual reason for tonsillectomy. The patients were treated in 52 different hospitals. The median number of cases per hospital was 27.5. The median duration of bilateral tonsillectomy was 24 minutes.

**Table 1:** Patients’ characteristics (n = 2556).

**Measure**	**Absolute**	**Relative**
Gender		
Male	1008	39.4%
Female	1537	60.1%
Unknown	11	0.4%
Age groups		
Children	165	6.5%
Adults	2177	85.2%
Unknown	214	8.4%
ASA status		
1	1326	51.9%
2	772	30.2%
3	37	1.4%
4	1	0.003%
Unknown	420	16.4%
Chronic pain prior to surgery		
No	1842	72.1%
Yes	513	20.1%
Unknown	201	7.9%
Type of surgery		
Tonsillectomy alone	2074	81.1%
Tonsillectomy plus other surgery	482	18.9%
	**mean ± SD**	**Median, range**
Age, mean ± SD, years	29.4±12.6	25, 4–85
Duration of surgery, mean ± SD, min	27±16	24, 2–190
Duration of surgery, if only tonsillectomy, mean ± SD, min	27±15	24, 4–172
If chronic pain, NRS*, mean ± SD	4.9±2.4	5, 0–10
Children, body weight, kg	38.7±21.72	29, 14–110
Children, BMI, kg/m^2^	26.7±10.9	22.5, 13–64
Number of cases per hospital	48.7±53.2	27.5, 1–224

*10-point numeric rating scale, higher numbers are indicating more pain (0 = no pain; 10 = maximal pain)

### QUIPS: Process parameter

S2 Table gives a detailed overview about the perioperative and postoperative measures applied as prevention or treatment of postoperative pain. Tonsillectomy was always performed in general anaesthesia. Analgesic and co-analgesic drugs were given in standard dosages. The predominant sedative for premedication was midazolam in 42% of the cases. Metamizole was the most frequently used non-opioid (18%) and piritramide the most frequent given opioid (34%) in the recovery room. Back on ward, 42% of patients received metamizole as non-opioid, followed by acetaminophen in 16% and ibuprofen in 14% of the cases, respectively. The most common opioid on ward was oxycodone (28%) followed by piritramide (5%). About half of the patient received a cold pack on ward (55%). Individual prescriptions of pain therapy were found in 74% of the charts. A documentation of patient’s pain in the chart was found in 66% of all cases.

### QUIPS: Outcome parameter

**Fig 2:**
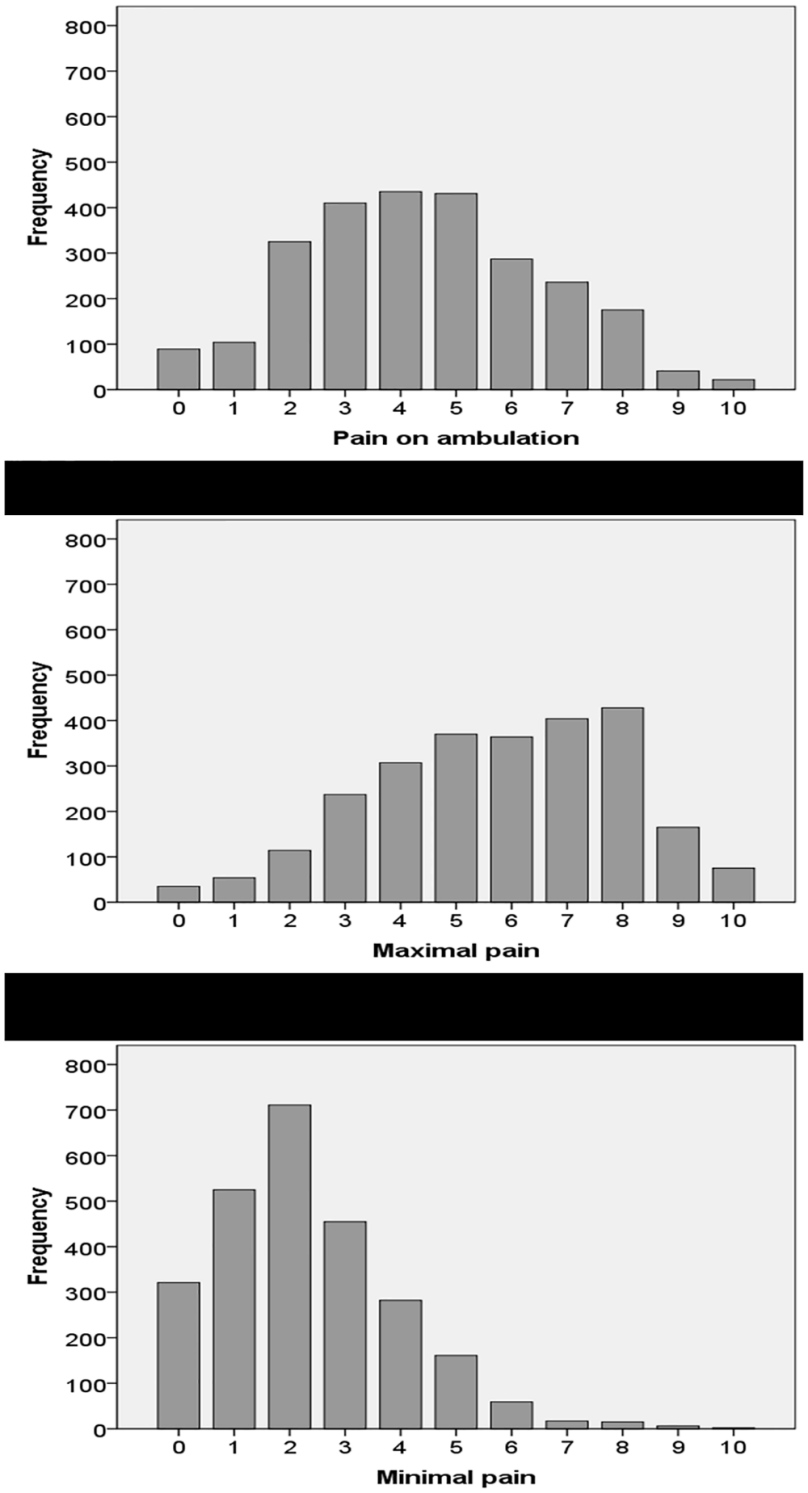
Distribution of the NRS scores for pain on ambulation (pain during movement), maximal pain, and minimal pain on the first postoperative day after tonsillectomy.

The results of QUIPS and QUIPSI questionnaires concerning patient-reported outcome parameters on the first postoperative day after tonsillectomy are presented in Table 2. The variability of the experienced pain intensity after tonsillectomy between patients is presented in Fig 2. Overall, the pain during movement, maximal pain and minimal pain were 4.4±2.2, 5.8±2.2, 2.3±1.7 (NRS), respectively. The three pain scores were highly correlated to each other: Pain during movement was correlated to maximal pain (r = 0.720; p<0.001) and minimal pain (r = 0.626; p<0.001). Maximal pain was correlated to minimal pain (r = 0.542; p<0.001). Maximal pain scores were not different between children (5.5±2.6) and adults (5.7±2.2). Three out of four patients (75%) reported to have received a general preoperative pain counselling but only 14% reported to have received a special counselling about postoperative pain and its management. The predominant pain-related functional interference was impaired breathing/coughing (74%), followed by impaired sleep (54%). The most frequent side effect was drowsiness (61%). The satisfaction with the pain management was moderate to good with 7.5±1.9 (NRS).

**Table 2:** Outcome parameters (n = 2556).

Measure	Absolute number of patients or mean ± SD	Relative number of patients or Median, range
**Numeric rating scale**		
Pain during movement, NRS*	4.4±2.2	4, 0–10
Maximum pain intensity, NRS*	5.8±2.2	6, 0–10
Minimum pain intensity, NRS*	2.3±1.7	2, 0–10
Satisfaction with pain therapy, NRS*	7.5±1.9	8, 0–10
**Pain-related interferes**		
Impaired mobility		
Yes	482	18.9%
No	1894	74.1%
Unknown	180	7.0%
Impaired breathing/coughing		
Yes	1647	64.4%
No	899	35.2%
Unknown	10	0.4%
Impaired sleep		
Yes	1384	54.1%
No	1160	45.4%
Unknown	12	0.5%
Impaired mood		
Yes	826	32.3%
No	1546	60.5%
Unknown	184	7.2%
**Pain therapy related side-effects**		
Tiredness/drowsiness		
Yes	1551	60.7%
No	989	38.7%
Unknown	16	0.6%
Nausea		
Yes	756	29.6%
No	1787	69.9%
Unknown	13	0.5%
Vomiting		
Yes	338	13.2%
No	1849	72.3%
Unknown	369	14.4%
**Other outcome measures**		
Pain counselling		
Yes, in general	1906	74.6%
Yes, specific	353	13.8%
No	258	10.1%
Unknown	39	1.5%
Desire for pain medication		
Yes	533	20.9%
No	1986	77.7%
Unknown	37	1.4%

*numeric rating scale (NRS) from 0 (no pain) to 10 (maximal pain)

### Factors associated with higher maximal pain intensity

**Fig 3:**
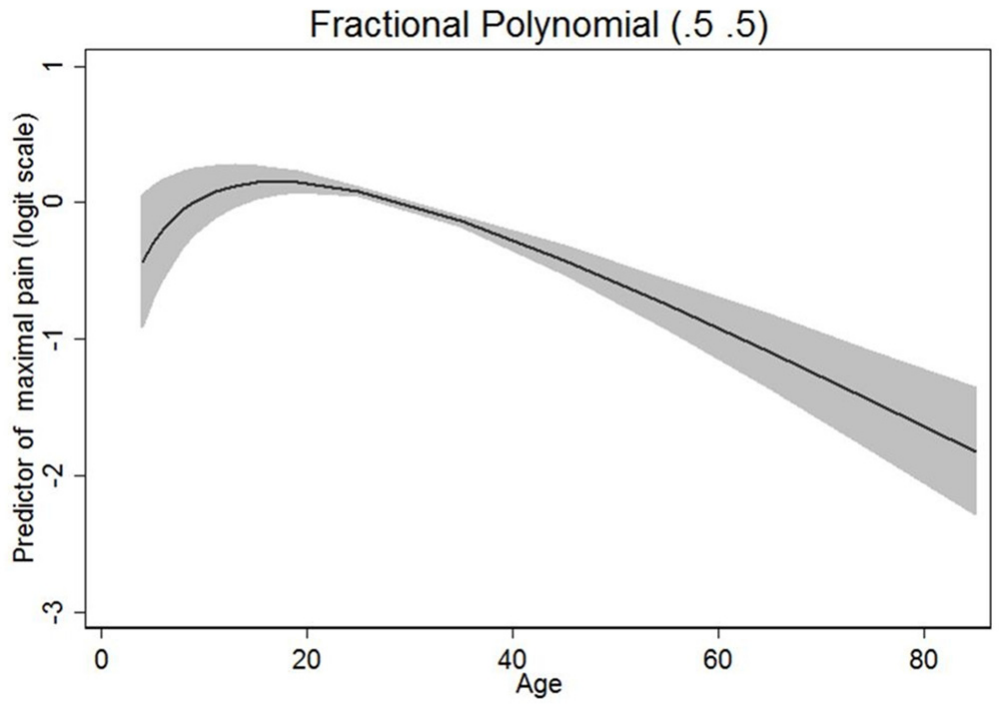
Functional relationship between age and maximal pain score based on fractional polynomials. The corresponding transformations of age are given by sqrt(age)-1.715 and by sqrt(age)*ln(age)-1.85. The predicted results are shown on the scale of the linear predictor together with a 95% confidence interval.

By univariable analysis, several patient and process factors were significantly (p<0.05) associated with higher experienced maximal pain (S3 Table). These factors were included in the multivariable analysis (see below). The effect of age on pain was not linear as indicated by Fig 3 and established using fractional polynomials: Maximal pain was in general high in underage patients and increased to a peak at the age of 18 to 20 years. From the age of ≥20 years on, maximal pain after tonsillectomy continuously decreased. All measured pain-related interference items (impaired mobility, breathing, sleep, mood) were associated with more maximal pain (all p<0.001). All measured side-effect symptoms (drowsiness, nausea, vomiting) were more frequently seen in patients with high maximal pain intensity after tonsillectomy (all p<0.001).

The multivariable analysis on independently associated parameters on maximal pain intensity revealed that female gender (Odds ratio [OR] = 1.33; 95% confidence interval [CI] = 1.12–1.58), age <20 years (OR = 1.56; CI = 1.27–1.91), no specific pain counselling (OR = 1.78; CI = 1.37–2.32), chronic pain (OR = 1.34; CI = 1.10–1.64), and receiving opioids in recovery room (OR = 1.89; CI = 1.55–2.32) or on ward (OR = 1.79; CI = 1.42–2.27) were independent predictors for higher experienced postoperative pain (Table 3). Furthermore, pain related impaired mobility (OR = 1.45; CI = 1.14–1.85), impaired breathing (OR = 2.27; CI = 1.87–2.77), impaired sleep (OR = 2.807; CI = 2.32–3.38), and impaired mood (OR = 2.71; CI = 2.21–3.33) were independently associated with higher maximal pain (Table 4). Drowsiness (OR = 1.94; CI = 1.62–2.33) and nausea (OR = 1.60; CI = 1.25–2.03; were independently associated with higher maximal pain, too (Table 4).

**Table 3:** Independent associations between baseline and process parameters and postoperative maximal pain.

Measure	OR	95% CI lower	95% CI upper	*P* value
Gender				
Male	1			
Female	1.330	1.124	1.575	**0.0010**
Age (years)				
Age≥20	1			
Age<20	1.560	1.273	1.913	**<0.001**
Specific pain counselling				
Yes	1			
No	1.782	1.370	2.316	**<0.001**
Chronic pain				
No	1			
Yes	1.340	1.097	1.636	**0.0041**
Recovery room, opioid				
No	1			
Yes	1.893	1.548	2.315	**<0.001**
Ward, non-opioid				
No	1			
Yes	1.791	1.415	2.266	**<0.001**

Multivariable ordinal logistic regression for outcome parameter maximal pain adjusted for gender, chronic pain, pain counselling, premedication, preoperative sedative, opioid usage in the recovery room, opioid usage on ward and age; numeric rating scale (NRS) from 0 (no pain) to 10 (maximal pain); OR = odds ratio, CI = confidence interval.

**Table 4:** Independent associations between pain-related interferes and pain therapy side effect and more postoperative maximal pain (>median NRS 6).

Measure	OR	95% CI lower	95% CI upper	*P* value
**Model 1: Pain-related interferes**				
Impaired mobility				
No	1	1		
Yes	1.449	1.137	1.846	**0.003**
Impaired breathing				
No	1	1		
Yes	2.274	1.865	2.773	**<0.001**
Impaired sleep				
No	1	1		
Yes	2.797	2.316	3.377	**<0.001**
Impaired mood				
No	1	1		
Yes	2.714	2.212	3.329	**<0.001**
**Model 2: Pain therapy related side-effects**				
Drowsiness				
No	1	1		
Yes	1.944	1.622	2.328	**<0.001**
Nausea				
No	1	1		
Yes	1.595	1.254	2.029	**<0.001**
Vomiting				
No	1	1		
Yes	0.937	0.695	1.263	0.669

Multivariable binary logistic regression for the dichotomized outcome parameter maximal pain (Maximal pain <median NRS 6 versus >median NRS 6); numeric rating scale (NRS) from 0 (no pain) to 10 (maximal pain); OR = odds ratio, CI = confidence interval.

**Fig 4:**
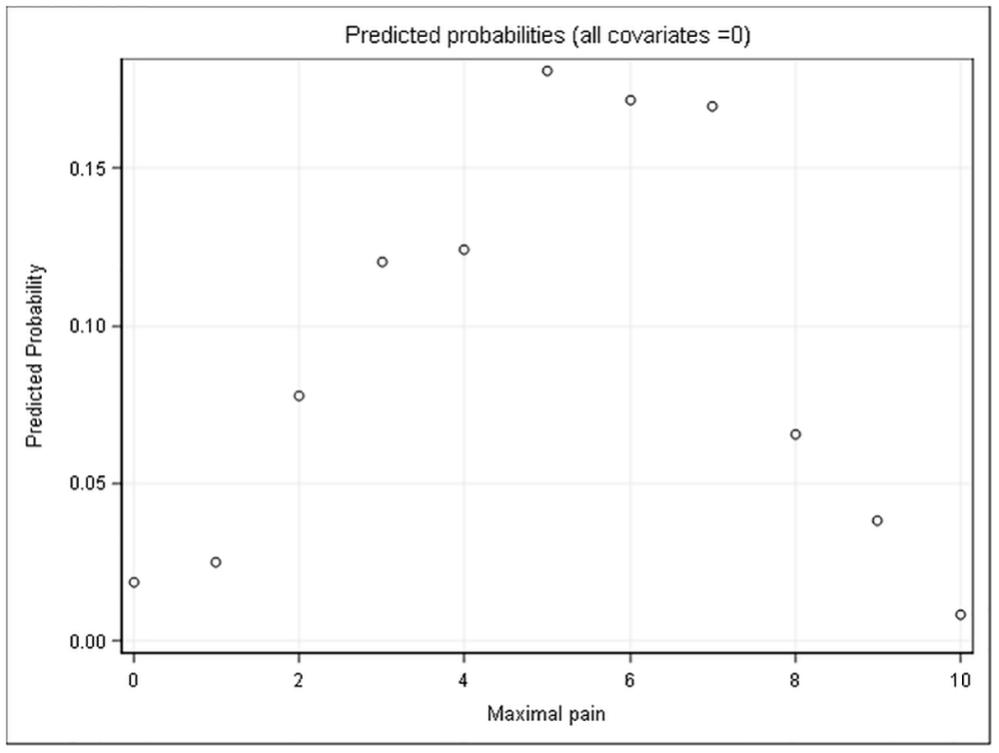
Estimated probability for each item of the maximal pain score showing that most patients will experience a maximal pain NRS score of 5, 6, or 7 on the first postoperative day after tonsillectomy.

**Fig 5:**
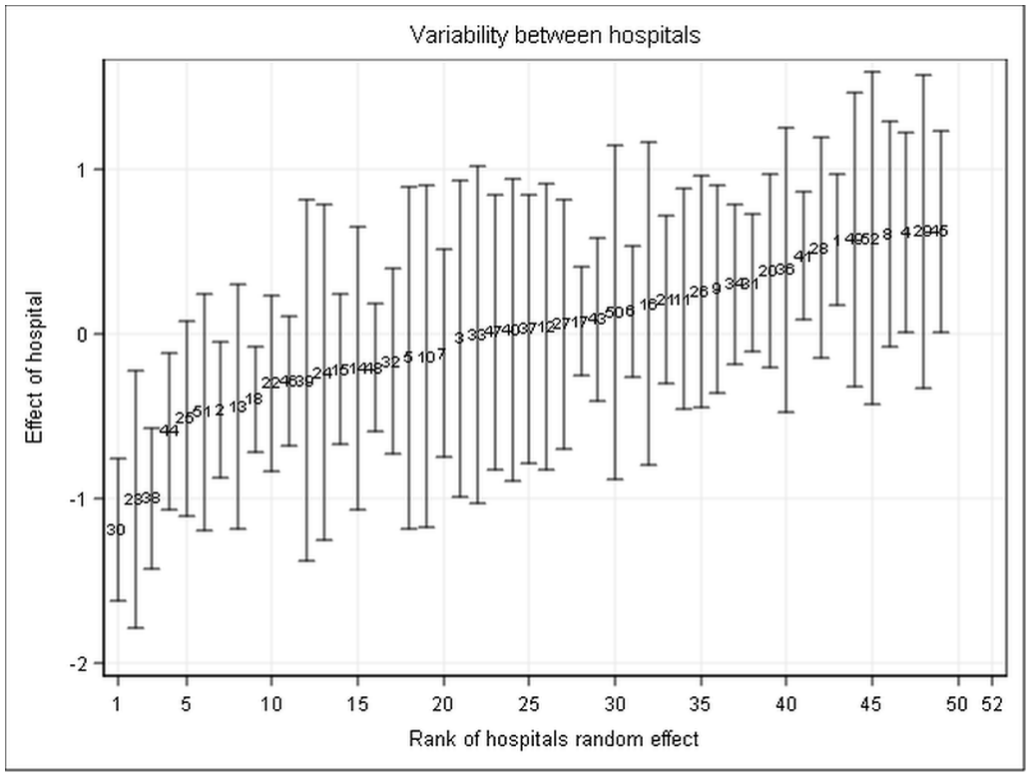
Ranking of hospital specific random effects estimates together with a 95% confidence interval. Even after adjustment to baseline and process parameters there still is a high variability of experienced pain between hospitals. Each bar and code number represent one of the 52 hospitals.

The ordinal logistic regression model was used to estimate the probability of the respective pain scores. The highest estimated probability was found for a maximal pain NRS of 5 the day after tonsillectomy (Fig 4). Our analysis showed that a high variability of the experienced maximal pain scores of the patients between the 52 participating hospitals remained even after adjustment to all statistically important baseline and process parameters (as listed in Tables 1 and 2; heterogeneity variance equal to 0.31). Fig 5 shows the ranked estimates of the random effects indicating the individual hospital’s deviation from the overall mean.

## Discussion

This large prospective study showed that postoperative pain after tonsillectomy was high with a median NRS score of 6. The study revealed that younger age (especially at the age of 18 to 20 years), female gender, and preoperative chronic pain were independently associated with higher postoperative pain at the first day after tonsillectomy. Furthermore, patients with higher postoperative pain had received significantly more opioids in the recovery room and on ward prior to the pain intensity assessment. More postoperative pain was related to more pain-related side effects in terms of impaired mobility, impaired breathing, impaired sleep, and impaired mood. It is important to notice that even after adjustment to the above mentioned predictors the variability of the experienced maximal pain remained high within a hospital and especially between different hospitals.

Strengths of this study are the large sample size and the standardized assessment of process and outcome parameters. The study measured pain intensity in German hospitals with almost exclusively Caucasian (white) patients. The German cultural setting and ethnic background might have influence on pain perception and pain treatment although the role of these factors and association with postoperative pain are discussed controversially [18–20]. Preoperative psychological factors and their influence on postoperative pain after tonsillectomy were not assessed. So far only one prospective cohort trial with 217 patients has explored the role of psychological factors on postoperative pain after otolaryngological surgery (including 16 patients with pharyngeal surgery not specified more precisely): Sommer et al. showed that pain catastrophizing (but not expected pain, or short-term fear) was an independent predictor for increased postoperative pain [21]. There is no international standard for cut-off points for pain intensity measurements. The multivariable analysis of the present study focussed on severe pain (NRS less than 6 versus NRS of 6 or higher) as here the need for better pain management is more than obvious. Nevertheless, the results cannot be directly referred to patients with moderate postoperative pain. In all of the patients, tonsillectomy was performed by traditional cold dissection. Therefore, the study may not reflect the situation for other tonsillectomy methods, but it should be mentioned that there is evidence that cold dissection seems to produce even less pain than other techniques [22]. In the present study no patient received a perioperative local infiltration of the tonsillar bed or topical local anaesthetics. Hence, the effect of local anaesthetics could not be analysed. There is some evidence that local anaesthetics seem to provide at least a modest reduction of post-tonsillectomy pain [23]. Finally, as in other non-randomized clinical trials, associations between outcome and any other factors have to be interpreted carefully and may not be causative. It was shown that patients with higher pain scores have received more often opioids in the recovery room and on ward. This finding can be interpreted that patients with higher pain intensities received more frequently opioids. Nevertheless, pain was often not sufficiently released. Potential explanations comprise insufficient dosing due to opioid-related effects or inadequate training of staff, or non-responding of tonsillectomy-related pain.

It cannot be excluded that participation at the QUIPS/QUIPSI registry per se indicates that the participating hospitals pay more attention on postoperative pain management than other sites. Therefore, the presented data may not be representative for Germany or other countries. Nevertheless, our findings of high pain intensity in participating hospitals further underline the importance of improved pain management after tonsillectomy.

The present study confirmed data from a recent QUIPS analysis of 30 different surgical procedures (including 332 adult patients with tonsillectomy): Gerbershagen et al. also showed that younger age, female gender, and preoperative chronic pain were associated with higher postoperative pain [12]. We can confirm now these results for tonsillectomy patients a) in a much larger cohort, b) even after adjustment for the postoperative pain treatment, and c) also for children and adolescents. Interestingly, the effect of age was not linear. Severe pain in underage patients, the peak of maximal pain in patients 18 to 20 years of age followed by a now linear decrease of pain with increasing age might be a specific characteristic of post-tonsillectomy pain. Thus for ease of interpretation we decided to dichotomise age at year 20. Then again, we are not aware of any other prospective study on postoperative pain including children and adults for the same type of surgery. If this non-linear effect also occurs after other types of surgery or not and the underlying reasons should be explored in further studies. It has to be emphasized that this non-linear effect would not have been noticed if only children or only adults would have been investigated. The applied methodology allowed a standardized analysis of postoperative pain independent of the patient’s age. Tonsillectomy is a surgery often performed in children and adults. Therefore it was rationale to analyse children and adults together.

Preoperative chronic pain but not younger age or female gender was a robust predictor of more postoperative pain after different types of surgery in systematic reviews and studies using other methods to measure pain than QUIPS/QUIPSI [12,24–27]. Although tonsillectomy is frequently used as postoperative pain model to evaluate new drugs or drug combinations, only a few prospective studies are published on predictors for postoperative pain after tonsillectomy. The already mentioned study by Sommer et al. in patients who underwent otorhinolaryngological surgery (no separate analysis of tonsillectomy patients; 7% pharyngeal surgery) showed that preoperative pain, pain catastrophizing, and anatomical site of surgery (oral cavity, pharynx, and larynx) were independent predictors for severe postoperative pain [21]. In accordance to the present study, preoperative pain counselling, no perioperative antibiotics (not analysed in the present study) and use of opioids on ward were associated with more postoperative pain in a univariate analysis performed in a previous prospective QUIPS single centre study on 65 patients after tonsillectomy [6]. Also two other small series in children using QUIPSI showed an association between (probably underdosed) opioid treatment on ward and more postoperative pain after otorhinolaryngological surgery [7,8].

Notwithstanding the present study reflects the clinical reality of health care beyond interventional studies. The present study suggests that many patients do not receive adequate or underdosed pain management after tonsillectomy. Several high-quality clinical practice guidelines based on results of controlled clinical trials for post-tonsillectomy pain management have been published for children and adults [4,28–30]. They all recommend maintaining adequate analgesia, especially on the first postoperative days. We strongly recommend first following such guidelines rather than looking for new pain drug combinations. The use and the effect of such guidelines have not been adequately studied yet. It may be worthwhile to train anaesthetists and otolaryngologists on a guideline or a fixed pain regime for tonsillectomy patients to analyse the effect on postoperative pain.

Not only related to tonsillectomy but in general for surgery with severe postoperative pain, more research is needed to delineate the role of specific aspects affecting sex and gender differences of experienced postoperative pain. In a previous QUIPSI study in children (4–18 years) who underwent paediatric otorhinolaryngological surgery (37% tonsillectomy) using female gender was a predictor for more maximal pain in univariate but not multivariate analysis [31]. A subanalysis of different age groups in the present study has shown that the gender effect was strong in adults (chi-square; p<0.001), moderate in adolescents 16 to 18 years of age (p = 0.046), and disappeared in any cohort of patients <16 years of age (p = 0.094). This suggests that hormonal changes (sex-related) and/or cultural differences in experiencing and reporting pain (gender role) may have an influence on pain assessment [32]. To differentiate these effects, on the one hand more laboratory studies on sex-related differences in patients with postoperative pain and on the other hand future studies applying cultural assessments and focussing on the influence of gender-specific education on pain experience are needed.

As it has been mentioned before psychological factors like pain catastrophizing may have a predictive value for postoperative pain after tonsillectomy [21]. But intrinsic psychological factors cannot explain why there also was a high variability of the pain scores between the different hospitals after adjustment of the detected predictors. There seem to be other extrinsic factors influencing the pain experience. Such factors may comprise hospital-related parameters like availability of acute pain services and standard operating procedures, training of staff, environmental influences, but also so-called soft factors like dedication and empathy of doctors and nurses. Moreover, socio-demographic and regional factors might have contributed to the observed differences. Good communication and empathy seem to play an important role in the preoperative and postoperative setting as it is well known for pain management in general [33–35]. Nevertheless, the intricacies of good communication in pain management are not well understood and much more research on communication concepts are needed [36].

## Conclusion

Even when less painful tonsillotomy will replace some more indications of classical tonsillectomy in the future, we will still be confronted with many cases of severe pain after tonsillectomy, underlining the need for better pain management. Beyond that tonsillectomy can be considered as a prototype of surgery followed by severe and short-term postoperative pain and pain management. The presented analysis showed a significant association between preoperative chronic pain, younger age, as well as female gender and more postoperative pain the day after tonsillectomy. Furthermore, a high variability of the pain scores was seen between the different hospitals even after adjustment to the detected predictors. Due to the observational nature of the study, causal inferences cannot be made. Further prospective trials would be essential stratifying pain management to postoperative pain predictors to demonstrate generalizability of our findings. Furthermore, other extrinsic, so far unknown factors influencing postoperative pain should be investigated.

## Supporting Information

S1 Table Parameters and coding. (DOCX)

S2 Table Process parameters in detail. (DOCX)

S3 Table Association between patients’ characteristics and process parameters with pain intensity. (DOCX)
