# Supplementary material for: Inter-Hospital Variability of Postoperative Pain after Tonsillectomy: Prospective Registry-Based Multicentre Cohort Study
Source: PLoS One. 2016 Apr 27;11(4):e0154155. doi: 10.1371/journal.pone.0154155 (PMC4847852; doi:10.1371/journal.pone.0154155)
Supplement: S1 Table — (DOCX) [file pone.0154155.s001.docx]

**S1 Table**

**S1 Table**. Parameters and coding.*

| **Parameter** | **Coding** |
| --- | --- |
| ID | consecutive number |
| inclusion | yes, no |
| gender | female, male |
| age | in years, at the time of surgery |
| ASA status | 1, 2, 3, 4 |
| first postoperative day | date (day, month, year) |
| begin of surgery | time (hours, minutes) |
| end of surgery | time (hours, minutes) |
| OPS-codes | coding of up to 5 OPS-codes |
| Tonsillectomy or Tonsillectomy plus other procedures | yes, no |
| pain counselling | yes, in general; yes, specific; no |
| pain during movement | NRS 0-10 |
| maximal pain | NRS 0-10 |
| minimal pain | NRS 0-10 |
| impaired mobility, | yes, no |
| impaired breathing | yes, no |
| impaired sleep | yes, no |
| impaired mood | yes, no |
| drowsiness | yes, no |
| nausea | yes, no |
| vomiting | yes, no |
| desire for pain medication | yes, no |
| satisfaction with pain management | NRS 0-15 |
| chronic pain prior to surgery | yes, no |
| chronic pain prior to surgery | NRS 0-10 |
| type of anaesthesia | general, regional, general and regional, local anesthesia only |
| regional anaesthesia | paravertebral, peripheral |
| premedication, sedative | no, midazolam, tranxilium, diazepam, promethazine, Haloperidol |
| premedication, non-opioid | no, acetaminophen p.o., acetaminophen i.v., acetaminophen i.m., acetaminophen supp., metamizole p.o., metamizole i.v., metamizole i.m., metamizole supp., ibuprofen p.o., ibuprofen supp., diclofenac p.o., diclofenac i.v., diclofenac i.m., diclofenac supp., celecoxib p.o., parecoxib i.v., eterocoxib p.o., gabapentin p.o., pregabalin p.o. |
| premedication, opioid | tramadol p.o., tramadol retard, tramadol i.v., tramadol i.m., tramadol supp., tramadol sc, pethidine p.o., pethidine retard, pethidine i.v., pethidine i.m., pethidine supp., pethidine sc, piritramide i.v., piritramide i.m., piritramide sc, morphine p.o., morphine retard, morphine i.v., morphine i.m., morphine supp., morphine ac, oxycodone p.o., oxycodone retard, oxycodone i.v., oxycodone i.m., oxycodone sc, fentanyl p.o., fentanyl i.v., fentanyl i.m., fentanyl sc, fentanyl trans, sufentanil i.v., sufentanil i.m., sufentanil sc, tilidine/(naloxone) p.o., tilidine/(naloxone) retard, , oxycodone/(naloxone) p.o., oxycodone/( naloxone) retard, hydromorphone p.o., hydromorphone retard, hydromorphone i.v., hydromorphone i.m., hydromorphone sc |
| intraoperatively: local anaesthesia | yes, no |
| Initiation of surgery: | no, remifentanil, clonidine, ketamine |
| recovery room, non-opioid | no, acetaminophen p.o., acetaminophen i.v., acetaminophen i.m., acetaminophen supp., metamizole p.o., metamizole i.v., metamizole i.m., metamizole supp., ibuprofen p.o., ibuprofen supp., diclofenac p.o., diclofenac i.v., diclofenac i.m., diclofenac supp., celecoxib p.o., parecoxib i.v., eterocoxib p.o., gabapentin p.o., pregabalin p.o. |
| recovery room, opioid | tramadol p.o., tramadol retard, tramadol i.v., tramadol i.m., tramadol supp., tramadol sc, pethidine p.o., pethidine retard, pethidine i.v., pethidine i.m., pethidine Supp., pethidine sc, piritramide i.v., piritramide i.m., piritramide sc, morphine p.o., morphine retard, morphine i.v., morphine i.m., morphine supp., morphine ac, oxycodone p.o., oxycodone retard, oxycodone i.v., oxycodone i.m., oxycodone sc, fentanyl p.o., fentanyl i.v., fentanyl i.m., fentanyl sc, fentanyl trans, sufentanil i.v., sufentanil i.m., sufentanil sc, tilidine/(naloxone) p.o., tilidine/(naloxone) retard, , oxycodone/(naloxone) p.o., oxycodone/( naloxone) retard, hydromorphone p.o., hydromorphone retard, hydromorphone i.v., hydromorphone i.m., hydromorphone sc |
| ward, non-opioid | no, acetaminophen p.o., acetaminophen i.v., acetaminophen i.m., acetaminophen supp., metamizole p.o., metamizole i.v., metamizole i.m., metamizole supp., ibuprofen p.o., ibuprofen supp., diclofenac p.o., diclofenac i.v., diclofenac i.m., diclofenac supp., celecoxib p.o., parecoxib i.v., eterocoxib p.o., gabapentin p.o., pregabalin p.o. |
| ward, opioid | tramadol p.o., tramadol retard, tramadol i.v., tramadol i.m., tramadol supp., tramadol sc, pethidine p.o., pethidine retard, pethidine i.v., pethidine i.m., pethidine supp., pethidine sc, piritramide i.v., piritramide i.m., piritramide sc, morphine p.o., morphine retard, morphine i.v., morphine i.m., morphine supp., morphine ac, oxycodone p.o., oxycodone retard, oxycodone i.v., oxycodone i.m., oxycodone sc, fentanyl p.o., fentanyl i.v., fentanyl i.m., fentanyl sc, fentanyl trans, sufentanil i.v., sufentanil i.m., sufentanil sc, tilidine/(naloxone) p.o., tilidine/(naloxone) retard, , oxycodone/(naloxone) p.o., oxycodone/( naloxone) retard, hydromorphone p.o., hydromorphone retard, hydromorphone i.v., hydromorphone i.m., hydromorphone sc |
| ward, physical pain therapy | cold pack, |
| ward, individual pain therapy instruction | yes, no |
| ward, pain documentation in patient chart | yes, no |

*Pain therapy measures and drugs were only registered when applied before the patient filled out the QUIPS/QUIPSI questionnaire.

NRS = numeric rating scale; p.o. = per os; i.v. = intravenous; i.m. = intramuscular; supp. = suppository; sc = subcutaneous; trans = transcutaneous.
