# Supplementary material for: Inter-Hospital Variability of Postoperative Pain after Tonsillectomy: Prospective Registry-Based Multicentre Cohort Study
Source: PLoS One. 2016 Apr 27;11(4):e0154155. doi: 10.1371/journal.pone.0154155 (PMC4847852; doi:10.1371/journal.pone.0154155)
Supplement: S2 Table — (DOCX) [file pone.0154155.s002.docx]

**S2 Table**

| **S2 Table.** Process parameters in detail (n=2556).* | |
| --- | --- |
| **Measure** | **Absolute (Relative)** |
| Premedication  Diazepam  Midazolam  Tranxilium  Diclofenac  Celecoxib  Etoricoxib  Ibuprofen  Metamizole  Naproxen  Acetaminophen  Oxycodone  Piritramide  Sedative  Non-opioids*  Opioids*  No premedication  Combination of non-opioid and opioid  Only non-opioid  Only opioid  Neither non-opioid nor opioid | 155 (6.1%)  1081 (42.3%)  296 (11.6%)  96 (3.8%)  99 (3.9%)  219 (8.6%)  113 (4.4%)  7 (0.3%)  6 (0.2%)  5 (0.2%)  465 (18.2%)  5 (0.2%)  1532 (40.1%)  544 (21.3%)  470 (18.4%)  1024 (40.1%)  116 (4.5%)  428 (16.7%)  354 (13.8%)  1658 (64.9%) |
| Induction of anaesthesia  Clonidine  Ketamine  Remifentanil | 142 (5.5%)  11 (0.4%)  935 (36.6%) |
| During surgery  Dexamethasone  Alfentanil  Remifentanil  Sufentanil  Clonidine  Ketoprofen  Metamizole  Acetaminophen  Fentanyl  Nalbuphine  Piritramide  Non-opioids*  Opioids*  Combination of non-opioid and opioid  Only non-opioid  Only opioid  Neither non-opioid nor opioid | 58 (2.3%)  65 (2.5%)  28 (1.1%)  14 (0.5%)  24 (0.9%)  1 (0.04%)  73 (2.8%)  76 (3.0%)  16 (0.6%)  5 (0.2%)  48 (1.9%)  116 (4.5%)  149 (5.8%)  106 (4.1%)  10 (0.4%)  43 (1.7%)  2397 (93.8%) |
| Intraoperative infiltration of the tonsillar bed with local anaesthetics | 0 (0%) |
| In recovery room  Clonidine  Dexamethasone  Diclofenac  Ibuprofen  Metamizole  Acetaminophen  Parecoxib  Morphine  Nalbuphine  Oxycodone  Pethidine  Piritramide  Tramadol  Non-opioids*  Opioids*  Combination of non-opioid and opioid  Only non-opioid  Only opioid  Neither non-opioid nor opioid | 26 (1.0%)  1 (0.04%)  9 (3.5%  9 (3.5%)  456 (17.8%)  109 (4.3%)  12 (0.5%)  81 (3.2%)  7 (0.3%)  129 (5.1%)  10 (0.4%)  869 (34.0%)  1 (0.04%)  667 (26.1%)  1195 (46.8%)  486 (19.0%)  181 (7.1%)  709 (27.7%)  1180 (46.2%) |
| On ward  Clonidine  Celecoxib  Diclofenac  Etoricoxib  Ibuprofen  Metamizole  Acetaminophen  Fentanyl  Hydromorphone  Morphine  Oxycodone  Piritramide  Tilidine  Tramadol  Non-opioids*  Opioids*  Combination of non-opioid and opioid  Only non-opioid  Only opioid  Neither non-opioid nor opioid | 1 (0.04%)  107 (4.2%)  127 (5.0%)  297 (11.6%)  369 (14.4%)  1067 (41.7%)  398 (15.6%)  41 (1.6%)  1 (0.04%)  64 (2.5%)  721 (28.2%)  116 (4.5%)  50 (2.0%)  38 (1.5%)  1686 (66.0%)  705 (27.6%)  646 (25.3%)  1040 (40.7%)  59 (2.3%)  811 (31.7%) |
| On ward, cold pack  Yes  No  Unknown | 1407 (55.0%)  480 (25.4%)  669 (26.2%) |
| On ward, individual pain therapy instruction, n  Available  Not available  Unknown | 1896 (74.2%)  109 (4.3%)  551 (21.6%) |
| On ward, pain documentation in patient chart  Yes  No  Unknown | 1698 (66.4%)  305 (11.9%)  553 (21.6%) |

* Pain therapy measures and drugs were only registered when applied before the patient filled out the QUIPS/QUIPSI questionnaire.”
