# Supplementary material for: Inter-Hospital Variability of Postoperative Pain after Tonsillectomy: Prospective Registry-Based Multicentre Cohort Study
Source: PLoS One. 2016 Apr 27;11(4):e0154155. doi: 10.1371/journal.pone.0154155 (PMC4847852; doi:10.1371/journal.pone.0154155)
Supplement: S3 Table — (DOCX) [file pone.0154155.s003.docx]

**S3 Table**

| **S3 Table.** Association between patients’ characteristics and process parameters with pain intensity.* | | | |
| --- | --- | --- | --- |
| **Measure** | **Maximal pain**  **<median NRS 6** | **Maximal pain**  **>median NRS 6** | ***P* value** |
| **Baseline parameter** | | | |
| Year of treatment  <median year 2011  >median year 2011 | 638  639 | 640  636 | 0.921 |
| Gender  female  male | 718  553 | 817  454 | **<0.0001** |
| Age  <median 25 years  >median 25 years | 557  621 | 612  549 | **0.009** |
| Weight (in children)  <median 29 kg  >median 29 kg | 45  50 | 39  33 | 0.384 |
| Body mass index (in children)  <median 22.5 kg/m^2^  >median 22.5 kg/m^2^ | 45  49 | 38  33 | 0.472 |
| Chronic pain  no  yes | 940  224 | 900  289 | **0.003** |
| If chronic pain,  NRS (0-5)  NRS (6-10) | 138  84 | 187  102 | 0.554 |
| ASA status  1  >1 | 639  424 | 684  386 | 0.070 |
| Tonsillectomy  alone  in combination with other procedure | 1034  243 | 1037  239 | 0.847 |
| Duration of surgery  <median 24 minutes  >median 24 minutes | 617  632 | 644  583 | 0.125 |
| Surgical load  <median 27.5 cases  >median 27.5 cases | 133  1924 | 142  1062 | 0.717 |
| **Process parameter** | | | |
| Preoperative pain counselling  No  Yes | 107  1149 | 151  1107 | **0.004** |
| Specific preoperative pain counselling  No  Yes | 1053  203 | 1110  148 | **0.001** |
| Premedication, sedative  No  Yes | 544  733 | 480  796 | **0.010** |
| Premedication, non-opioid  No  Yes | 989  288 | 1022  254 | 0.102 |
| Premedication, opioid  No  Yes | 1025  252 | 1059  217 | 0.075 |
| Premedication, combination of non-opioid and opioid  No  Yes | 1218  59 | 1219  57 | 0.853 |
| Premedication  No  Yes | 460  817 | 392  884 | **0.005** |
| During surgery, non-opioid  No  Yes | 1206  71 | 1233  43 | **0.007** |
| During surgery, opioid  No  Yes | 1193  84 | 1212  64 | 0.091 |
| During surgery, combination of non-opioid and opioid  No  Yes | 1187  90 | 1206  70 | 0.104 |
| During surgery, pain medication  No  Yes | 1188  89 | 1207  69 | 0.101 |
| Recovery room, non-opioid  No  Yes | 936  341 | 951  325 | 0.478 |
| Recovery room, opioid  No  Yes | 769  508 | 589  687 | **<0.0001** |
| Recovery room, combination of non-opioid and opioid  No  Yes | 1045  232 | 1022  254 | 0.263 |
| Recovery room, pain medication  No  Yes | 660  617 | 518  758 | **<0.0001** |
| Ward, non-opioid  No  Yes | 468  809 | 401  875 | **0.005** |
| Ward, opioid  No  Yes | 1001  276 | 847  429 | **<0.0001** |
| Ward, combination of non-opioid and opioid  No  Yes | 1022  255 | 885  391 | **<0.0001** |
| Ward, pain medication  No  Yes | 447  830 | 363  913 | **<0.0001** |
| Ward, cold pack  No  Yes | 249  639 | 230  767 | 0.013 |
| Ward, pain therapy instruction  No  Yes | 51  904 | 58  990 | 0.848 |
| Ward, pain documentation  No  Yes | 126  826 | 179  870 | **0.017** |
| Desire for pain medication  No  Yes | 1149  105 | 834  428 | **<0.0001** |
| Satisfaction with pain therapy (NRS**)  <median NRS 8  >median NRS 8 | 418  761 | 771  428 | **<0.0001** |
| **Pain-related interferes** | | | |
| Impaired mobility  No  Yes | 1032  145 | 860  337 | **<0.0001** |
| Impaired breathing  No  Yes | 1032  145 | 860  337 | **<0.0001** |
| Impaired sleep  No  Yes | 798  473 | 359  911 | **<0.0001** |
| Impaired mood  No  Yes | 956  219 | 588  607 | **<0.0001** |
| **Pain therapy related side-effects** | | | |
| Drowsiness  No  Yes | 600  667 | 386  884 | **<0.0001** |
| Nausea  No  Yes | 970  299 | 814  457 | **<0.0001** |
| Vomiting  No  Yes | 958  140 | 888  198 | **<0.0001** |

*significant *P*-values in bold; **10-point numeric rating scale.
